# Smallholder farmers’ decisions to the combined use of soil conservation practices in Tiwa watershed, Northwest highlands of Ethiopia

**DOI:** 10.1016/j.heliyon.2021.e05958

**Published:** 2021-01-13

**Authors:** Ermias Debie

**Affiliations:** Department of Geography and Environmental Studies, Bahir Dar University, Ethiopia

**Keywords:** Farmers decisions, Combine use, Soil conservation practices, Ethiopia highlands

## Abstract

Soil erosion by water is a critical problem in the northwest highland of Ethiopia. The limited decision of farmers to the combined use of soil conservation practices is one of the main accelerating factors to soil erosion by water. The study aimed to identify determinants influence farmers' decision to the combined use of vegetation stabilized terracing and composting under legume-cereal crop rotation on particular croplands in Tiwa watershed, northwest highlands of Ethiopia. The survey was conducted among randomly selected 155 household heads. Descriptive statistics and logistic regression models were used to identify the determinants. Farmland ownership status, plot distance from home, soil fertility status, and technical fitness of terraces were major influential factors for farmers’ decision to the combined use of vegetation stabilized terracing, compost, and legume-cereal crop rotation on a specific field. Therefore, to promote the combined use of soil conservation practices in the broad context, the policy should be recognized these institutional, technical, and plot-level factors influence farmers' decision to adopt over time.

## 1 Introduction

Soil erosion by water is a severe problem in the highlands of Ethiopia, where the subsistence farming system is the livelihood activity for the majority of smallholder farmers. The estimated annual soil loss due to erosion by water in Ethiopia is 1.5 billion tons, of which 50% occurs in croplands (Assefa and Bork, 2015). The upper blue Nile basin is estimated to generate an average soil loss rate of 27.5t ha^−1^yr^−1^, of which at least 10 % comes from gully erosion, and 26.7 % leaves Ethiopia (Haregeweyn et al., 2017). Soil loss could vary across the basin and watershed in response to different socioeconomic, institutional, plot level, and technical factors. For instance, at the watershed level of northwest highlands of Ethiopia, 91.6 Mg ha^−1^yr^−1^ soil (Bezuayehu and Sterk, 2010), and 19.2 Mg ha^−1^yr^−1^ soil (Mekuriaw et al., 2018) were lost due to soil erosion by water. Alemu and Melesse (2020) reported that 37 t ha^−1^yr^−1^ and 45 t ha^−1^ yr^−1^ soils were lost in conserved and adjacent non-conserved fields, respectively. The total volume of all rills was 22.8m^3^ha^-1^ yr^−1^ in non-conserved fields and 10.6 m^3^ ha^-1^ yr^-1^ in conserved fields, micro-watershed (Debie et al., 2019). Soil erosion is caused by overgrazing, population density, removing crop residues, intensive cultivation, and low nutrient application (Pimentel and Burgess, 2013; Haregeweyn et al., 2017). Clearing of vegetation covers and encroachment of cultivated fields in hilly and steep slopes results in increasing soil loss (Tadesse et al., 2017). Concentrated run-off entering from the uphill direction, terraces and drainage ditches damage, and improper practice of conservation practices were found to the major accelerating factors to soil erosion by the rill in the cultivated field (Debie et al., 2019).

The accelerated rate of soil degradation resulted in major ecological and socioeconomic problems on the agricultural lands of Ethiopia's highlands (Hurni et al., 2016). For example, increasing soil erosion severely limits the sustainable productivity of subsistence production under the crop-livestock mixed farming system (Gelagay and Minale, 2016; Teshome et al., 2016). The loss of soil organic carbon contributes to climate change results in low agricultural production and low resilience capacities of smallholder farmers (Georgise et al., 2019).

To avert the problems, indigenous soil conservation practices have been undertaken for centuries in the northwest highlands of Ethiopia (Monsieurs et al., 2015). The introduced soil conservation efforts have been carried out with no recognition of farmers' interest in decision-making processes (Debie, 2016; Teshome et al., 2016). The conservation efforts were carried out through the top-down approach of incentive-based food-for-work programs that favored to technical aspect with the typical intention of reducing soil erosion (Gebregziabher et al., 2016). The sustainable land management programs through the mobilization of uncompensated labor on the community level collaborated with government and development partners are carried out (FDRE, 2012). However, it is not implemented to reverse land degradation, promote farmers' incomes and food security, and protect ecosystem integrity and functions as set out in the objectives of the program (Haregeweyn et al., 2015).

The combined use of compost and terracing under the legume-cereals crop rotation (LCCR) system is unlikely in the highlands of Ethiopia despite efforts made through extension programs to scale up the practices (Haile et al., 2006). Combination of nutrient saving (such as, controlling erosion and recycling crop residues) and nutrient adding, such as applying compost should be promoted (Vanlauwe et al., 2011; Erkossa et al., 2018; Bekele and Negesse, 2019). This in turn important to scale up farmers’ adoption (Anley et al., 2007), to reduce water, soil, and nutrient losses at an acceptable level, and then to boost agricultural production in the farming systems of Ethiopia (Haile et al., 2006).

Vegetation stabilized terracing reduce run-off concentration and soil erosion and encourage the infiltration capacity of the soil (Morgan, 2005; Blanco and Lal, 2008). These principal roles should further supplement by agronomic practices like composting and LCCR (Morgan, 2005). Composting is enhanced soil fertility, structures, moisture retention, and erosion reduction (Eusuf Zai et al., 2008; Evanylo et al., 2008). It enables to reduce the financial risk of buying chemical fertilizers on credit (Kassie et al., 2009). Under LCCR, composting is preferable to maintain nitrogen status and soil organic carbon sequestration in the soil (Degu et al., 2019; Bossio et al., 2020).

The decisions of farmers to manage soil largely depend on intermingled institutional, socioeconomic, technical, and plot-level factors (Sileshi et al., 2019; Melese et al., 2019). Numerous studies conducted on determinants influencing the adoption of introduced physical soil and water conservation practices in Ethiopia highlands (Amsalu and De Graaff, 2007; Anley et al., 2007; Shiferaw et al., 2008; Teshome et al., 2016; Meseret and Amsalu, 2017; Asmame and Abegaz, 2017; Asfaw and Neka, 2017; Mekuriaw et al., 2018; Sileshi et al., 2019; Mengistu and Assefa, 2019; Melese et al., 2019). The study aimed to identify determinants influence farmers' decision to the combined use of vegetation stabilized terracing, composting, and LCCR in the Tiwa watershed northwest highlands of Ethiopia.

## 2 Materials and methods

### 2.1 Description of the study area

**Figure 1:**
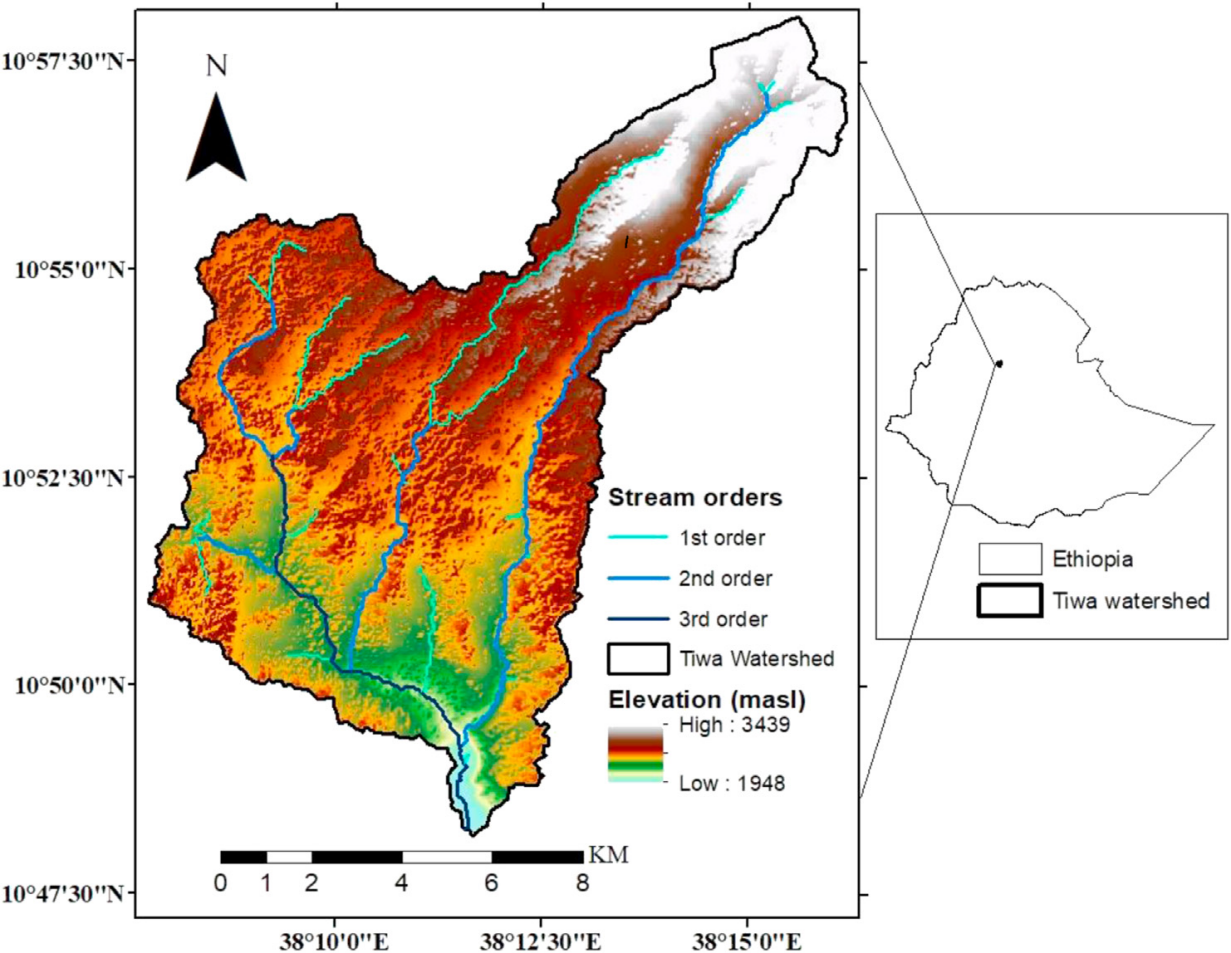
Location of the study site.

There is a prevalence of conservation efforts in the middle part of the Tiwa watershed for more than fifteen years. The study site was identified based on the cropping pattern and the status of soil conservation practice. The watershed lies between 38°7′44.227″ E_38°16′10.067″E longitudes and 10°48′12.071″N_10°58′2.239″ N latitudes (Figure 1). The geological characteristic of the watershed is categorized by the tarp series volcanic rock formed during the Cenozoic Era (Billi, 2015). The watershed is characterized by diverse topographic conditions. The major landforms in the selected site are characterized as gently sloping and slightly dissected undulating surfaces. Eutric Cambisols and Pellic Vertisols are largely distributed. The watershed in general falls within three agro-climatic zones (cool-moist, tepid-moist, and warm highlands) that are equivalent to the Ethiopian traditional agro-ecological zones of *Dega, Woina-Dega*, and *Kola,* respectively with the elevation ranges from 1,948 to 3,439 m.a.s.l (Figure 1). The middle part of the watershed is mainly situated in the tepid-moist agro-ecological zone, where high annual rainfall and moderate temperature are recorded (Hurni et al., 2016). The local climate is dominantly humid sub-tropic. Rainfall varies spatially from 1326.5 mm to 917.9 mm, where more than three-fourths percent of the total rainfall occurs in the summer season (from June to September). Wetlands, croplands and settlements, grasslands, shrub lands, and forests are the major land use/covers in the study watershed (Debie, 2016). Under the mixed farming systems of smallholder communities, tef (*Eragrostis tef*) and wheat (*Triticum vulgare*) are predominantly grown in the watershed. After that maize (*Zea mays*), barley (*Hordeum vulgare*), Niger seed (*Guizotia-abyssinica),* legume crops (horse beans (*Vicia faba*), pea (*Pisum sativum*)), barley (*Hordeum vulgare*), maize (*Zea mays*), and Niger seed (*Guizotia-abyssinica*) are produced. In the cultivated field, terracing (stone/soil bunds and *Fanya-juu*) stabilized with vegetative measures (like planted *Sesbania sesban* shrubs and natural grass), composting, legume-cereal crop rotation, and traditional ditches are largely practiced (Debie, 2016).

### 2.2 Methods of data collection

For this study, there were two stages of data collection procedures. In the first phase, field observations, informal discussions with ten farmers, and key informant interviews with five watershed committees and three development agents (DAs) were held. This phase of the pilot survey was important to obtain background information about a farming system, and practicing and adoption patterns of soil conservation practices. Based on the pilot information and empirical literature, structured questionnaires were prepared for socioeconomic, institutional, and technical and plot factors for the second phase of the household survey. In the survey, a random sample of 155 farming household heads (128 from terraces adopters and 27 from non-adopter) were involved. Three enumerators (grade 10 and above education level) were chosen to conduct the formal household survey. The researcher was provided training to all enumerators concerning the proper ways of administering questionnaires and collecting reliable data. The enumerators accompanied by close supervision of the researcher conducted face to face interviews with all the sampled farmers. Respondents were interviewed at home, on farmlands, and when available at the assembly area.

### 2.3 Methods of data analysis

The generated data were organized in tabular and diagrammatic form. The data were analyzed using descriptive statistics, and a binary logistic regression model by applying a statistical package for social scientists (SPSS, Version 21). Kolmogorov-Smirnov test was used to check the normality of the data generated from ≥50 samples (Ghasemi and Zahediasl, 2012). Chi-square test and independent t-test were employed to identify significant categorical and continuous variables respectively for further binary logistic regression analysis at P ≤ 0.05 and P ≤ 0.01 levels. A Chi-square test was used to test the statistical independence between two responses by using categorical independent variables. An independent t-test was used to test the statistical differences between the means of two response groups by using continuous independent variables. The correlation matrix was employed to check for high inter-correlations or multicollinearity among the predictor variables. The absolute value of the Pearson correlation coefficient of >0.7 among two or more predictors, indicates the presence of multicollinearity (Young, 2017). The binary logistic regression model, using maximum likelihood estimation, was employed to identify determinants on farmers' decision to the combined use of soil conservation practices. The model was used to analyze the cause-effect association between dichotomous dependent categories (adopter/non-adopter or user/non-user) and independent variables, such as non-categorical and categorical (Pallant, 2000).

### 2.4 Binary logistic regression model specification

The binary logistic regression model empowers one to select the predictive model for dichotomous dependent variables. It describes the relationship between a dichotomous response variable and a set of explanatory variables (predictors). For this study, the binary logistic regression model was used to scrutinize the predictors on the probability of the response variables (adopter/user) Yij, and Y_ij_ takes a value of 1 if the households adopt/use conservation practices and 0 otherwise (see Table 1). Let us denote the proportion of adopter/user by$p\left(Y_{i}=1\right)=\pi_{ij}$, and the proportion of non-adopter or non-user by $p\left(Y_{i}=1\right)=1-\pi_{ij}$ with the assumption of Yi ~ Bernoulli (πi). Besides, **X**_nx(k+1)_ denote the single level binary logistic regression data design matrix of k predictor, for the response variables adoption status and β_(k+1)x1_ be a vector of unknown coefficients of the covariates and intercept and given as:

(1) $$X=\left[\begin{array}{lllll} 1 & x_{11} & x_{12} & ... & x_{1k}\\ 1 & x_{21} & x_{22} & ... & x_{2k}\\ \text{M} & \Lambda & \Lambda & \Lambda & \text{M}\\ 1 & x_{n1} & x_{n2} & ... & x_{nk} \end{array}\right]\;\beta =\left[\begin{array}{l} \beta_{o}\\ \beta_{1}\\ \text{M}\\ \beta_{k} \end{array}\right]$$

**Table 1:** Definition, measurement, and hypothesis of dependent and independent variables used in empirical models.

Acronym	Definition and Measurement of Dependent Variables
TERRACE	Terracing measures adoption: 1 if farmers adopt terrace stabilized with vegetation measures, 0 otherwise
COMP	Compost use: 1 if farmers regularly use in any plot,0 otherwise
LCCR	Legume-cereals crop rotation practice: 1 if farmers often use in any plot, 0 otherwise
CUSCPs	The combined use of soil conservation practices: 1 if there is combined use of vegetative stabilized terracing, compost, and LCCR at the particular plot, 0 otherwise

Acronym	Definition and measurement of socioeconomic variables	Hypotheses
SEX∗	Gender of household head: 1 if male, 0 otherwise	Sex not likely influences farmers' decision to adopt soil conservation practices (SCPs)
AGE∗	Age of household head: age in years	Farmers age may not necessarily influence farmers' decision to adopt SCPs
PROLABSIZ∗	Productive labor size: in number	More productive labor size may significantly contribute to adopting SCPs
FAMSIZ∗	Family size: in number	Family size not likely influences farmers' decision to adopt SCPs
EDU∗	Education level: 1 if literate, 0 otherwise	The education level of the household head has more likelihood to adopt SCPs
FARMSIZE∗	Farmland holding size: Total farm size held per household in a hectare	Farm size has more likelihood to adopt SCPs
LIVHOLD∗	Livestock hold: in numbers	The size of livestock holding has more likelihood to adopt compost than others
USEDUNG	Use of animal dung for fuel in compost making time: 0 if yes, 1 otherwise	Using animal dung may negatively influence CUSCPs
OFFFARM∗	Off-farm income for fertilizer cost: 1 if no, 0 otherwise	The presence of off-farm income has no likelihood influence on the adoption of SCPs
BENAWAR∗	Adequate awareness of benefits of SCP:1 if yes, 0 otherwise	Adequate awareness of the benefits of SCPs has a positive likelihood of adoption decision
EXTN∗	Considerable extension contact of farmers with DAs: 1 if yes, 0 No	Extension contact likely positive significant effect on the adoption decision
FLOWNS	Ownership of field treated with SCP:1 if owns, 0 otherwise	Farmlands owners have more likelihoods of CUSCPs
PERDIST	Perceived distance to the home of field treated with SCP:1if nearby, 0 otherwise	Homestead and croplands that sited very close to residential area have more probability to treated by CUSCPs
PERFERT	Perceived fertility status of cropland treated with terraces: 1 if infertile, 0 otherwise	Perceived infertile status of cropland is more likely associated with farmers decision to CUSCPs
PERSLOP	Perceived slope categories of cropland treated with terraces: 0 if gentle, 1 otherwise	The gentle slope of particular croplands may negatively influence the adoption of vegetative stabilized terracing
TECHFIT	Technical fitness of terraces in arresting soil erosion by water, and appropriateness to local plow operation and crop efficiency: 1 if yes, 0 otherwise	Technical fitness terraces are more likely affected the adoption decision of terracing and CUSCPs

**Note**: For this study, FLOWNS, PERSLOP, PERFERT, PERDIST, and TECHFIT are common factors for the decision to adopt terracing and to CUSCP. USEDUNG was also a common factor for the decision to use compost and CUSCP. Moreover, ∗ stands for common predictor variables for all dependent variables (Terracing, composting, LCCR, and CUSCPs)*.*

Considering the descriptions given in Eq. (1), the logistic regression function can be defined as:

(2) $$\pi_{i}=\frac{\exp\left(\beta_{o}+\beta_{1}X_{i1}+\beta_{2}X_{i2}+...+\beta_{k}X_{ik}\right)}{1+\exp\left(\beta_{o}+\beta_{1}X_{i1}+\beta_{2}X_{i2}+...+\beta_{k}X_{ik}\right)}=\frac{\exp\left({X_{i}}^{\prime }\beta \right)}{1+\exp\left({X_{i}}^{\prime }\beta \right)}$$

where $\pi_{i}\;i=1,2,\ldots ..,n$ is the i^th^ probability of households use combined soil conservation practices given a set of predictors X. After algebraic manipulation, the multivariable logistic regression model can be written as in terms of an odds ratio (Eq. (3)) and logit link (Eq. (4)) for *i* = 1, 2,…, k as:

(3) $$\theta =\frac{P\left(y=1/Xi\right)}{1-p\left(y=1/Xi\right)}=\exp\left(\beta_{o}+\beta_{1}X_{1}+\beta_{2}X_{2}+...+\beta_{k}X_{k}\right)=\exp\left({X_{i}}^{\prime }\beta \right)$$

(4) $$\log\left(\frac{P\left(y=1/Xi\right)}{1-p\left(y=1/Xi\right)}\right)=\log\left(\frac{\pi_{i}}{1-\pi_{i}}\right)=\beta_{o}+\beta_{1}X_{1}+\beta_{2}X_{2}+...+\beta_{k}X_{k}={X_{i}}^{\prime }\beta$$

### 2.5 Parameter estimation and goodness of fit test

The logistic regression model uses maximum likelihood estimation (MLE) to estimate the unknown coefficients (parameters) that are included in the model. Hence, in this study, the maximum likelihood estimation technique was employed to estimate the unknown parameters of the model. The likelihood ratio (G^2^) test (log-likelihood test) was used to assess the overall fit of the fitted logistic regression model. And the Hosmer-Lemeshow test (a test procedure formulates under the null hypothesis that the model fits the data well, and the alternative is the model does not fit) was employed. Lastly, the Wald test was used to test the significance of individual logistic regression coefficients for each predictor. Besides, Akaike's information criterion (AIC) and Bayesian information criterion (BIC) were considered the model selection criteria.

## 3 Operational definitions and measurements of variables

For this study, adoption is defined as adopting and maintaining constructed terraces without any modification. Adapting is defined as adopting and maintaining constructed terraces with some modification through practicing alternative drainage ditches. Hence, farmers who adopted and adapted constructed terraces were considered adopters. Besides, farmers destroyed previous terraces from cropping fields with or without substituting by drainage ditches in the particular farming system were non-adopter. Based on the information generated from the sampled terraces adopters and non-adopters, further categorical dependent variables like users/non-users of compost, LCCR, and the combination of all three practices on the specific field were developed.

## 4 Results and discussion

### 4.1 Determinants of farmers decision to the combined use of soil conservation practices

Table 2 reveals significant differences between responses about farm size holding (FARMSIZE) to all types of conservation practices and productive labor size (PROLABSIZ) to terracing and composting. However, there was no significant mean difference between responses about age, sex, family size, livestock holding size, and the number of literate family members to all practices. These continuous variables were not incorporated for further logistic regression analysis.

**Table 2:** The statistical differences between the means of two response groups by using continuous independent variables.

Type of Practices	Categories of Respondents	Explanatory factors (t-test values)	
		FARMSIZE (in ha)	PROLABSIZ (in No.)
Terraces	Adopters (n = 128)	4.5^a^	5.8^a^
	Non-adopters (n = 27)		
Compost	Adopters (n = 135)	-1.97^b^	5.3^a^
	Non-adopters (n = 20)		
LCCR	users (n = 78)	7.5^a^	-
	Non- users (n = 77)		
CUSCP	users (n = 57)	3.7^a^	-
	Non-users (n = 98)		

Note: ^a^ and ^b^ indicate significance at p < 0.05 and p < 0.01, respectively.

Table 3 portrays significant differences between response categories of extension contacts (EXTN) to terracing and composting, and ownership status of cultivated fields (FLOWNS) to terracing and combined use of conservation practices (CUSCPs). Chi-square test results indicate a significant difference between categories of technical fitness of constructed terraces (TECHFIT) to terracing and CUSCPs. There were significant differences between categories of plot distance from home (PERFERT) to all practices and perceived fertility status of the plot (PERFERT) to CUSCPS.

**Table 3:** The statistical independence between two responses by using categorical independent variables.

Factors	Categories	adopter &non-adopter (*χ*^2^) of conservation practices			
		Terraces	Compost	LCCR	CUSCPs
PERSLOP	Gentle	17.6^a^	-	-	-
	Otherwise				
FLOWNS	owned	40.4^a^	-	-	13.63^a^
	rented				
EXTN	yes	10.1^a^	85.7^a^	-	-
	No				
PERDIST	nearby	24.1^a^	12.13^a^	23.14^a^	7.71^a^
	otherwise				
PERFERT	infertile	-	-	-	3.72^b^
	otherwise				
TECHFIT	yes	10.7^a^	-	-	10.99^a^
	No				

Note: ^a^, and ^b^ significant at p < 0.01 and 0.05 levels, respectively.

Besides, a significant difference was observed between perceived slope categories of a plot (PERSLOP) to terracing (Table 3). However, the differences between response categories were not significantly observed in sex, adequate awareness of benefits of conservation practices, off-farm activities, and use of dung for cooking fuel during compost making time. These socioeconomic variables were excluded from further analysis of the binary logistic regression model.

Results of the goodness of fit test in Table 4 reveal that the set of variables used as predictors in the binary logistic regression model fits. For instance, the omnibus tests of model coefficients of terraces (χ2 = 77.6, at df = 8 and Sig. p < 0.000), compost (χ2 = 97.54, at df = 5 and Sig. p < 0.000), LCCR (χ2 = 194, at df = 5 and Sig. p < 0.000) and CUSCPs (114.67, at df = 7 and Sig. p < 0.000) indicate the goodness of fit. This further interprets that result in a significant value at p < 0.05 gives an overall indicator of how well data fit the model. The overall correctly classification schemes in the model were 90.5% (98.1% for adopters and 55.9% for non-adopters of terracing), 95.2% (96.9% for adopters and 84.6% non-adopters of composting), 92.1% (93.6% for users and 90.5% for non-users of LCCR) and 91% (80.6% for users and 89.3% for non-users of CUSCPs). Results of correlation matrixes reveal that multicollinearity between the explanatory variables was not found to be a problem for the study. This is because the absolute value of the Pearson correlation coefficient was less than 0.7 (Young, 2017).

**Table 4:** Determinant influence farmers’ decision to the combined use of soil conservation practices.

Explanatory Variables	Terraces coefficient (*B*)	Compost coefficient (*B*)	LCCR coefficient (*B*)	CUSCPs coefficient (*B*)
PROLABSIZ	.625 (.252)^b^	1.1 (.39)^a^	-	-
FARMSIZE	.219 (.374)	-.838 (.46)^b^	2.145 (.604)^a^	-.389 (.317)
EXTN (1)	.995 (.559)^b^	4.893 (.91)^a^	-	-
FLOWNS (1)	1.84 (.66)^a^	-	-	2.64 (.92)^a^
PERDIST (1)	2.33 (.594)^a^	3.1 (1.34)^b^	3.217 (1.16)^a^	.983 (.477)^b^
PERFERT (1)	-	-089 (.75)	1.547 (.990)	1.624 (.65)^b^
TECHFIT (1)	.732 (.59)	-	-	2.1 (.68)^a^
Constant	-5.483 (1.22)^a^	-2.71 (1.3)^b^	-9.489 (2.234)^a^	-8.795 (1.44)^a^
-2 Log likelihood	100.536	53.86	67.963	131.1
Omnibus Tests of Model Coefficients	model – χ2 (77.6) at df = 8 and Sig.(p < 0.000)	model – χ2 (97.54) at df = 5 and Sig. (p < 0.000)	model – χ2 (194) at df = 5 and Sig.(p < 0.000)	model – χ2 (114.67) at df = 7 and Sig.(p < 0.000)
Correctly predicted^a^	90.5	95.2	92.1	91
Sensitivity^b^	98.1	96.9	93.6	80.6
Specificity^c^	55.9	84.6	90.5	89.3

Note

➢values of standard error (S.E.) are presented in a bracket.

➢^b^, and ^a^ denote statistical significance at the 5 % (p < 0.05), and 1 % (p < 0.01), respectively.

➢^a^ based on a 50-50 probability classification scheme.

➢^b^ correctly predicted adopters based on the 50-50 probability classification scheme.

➢^c^ correctly predicted non-adopters based on a 50-50 probability classification scheme.

➢CUSCPs –combine the use of soil conservation practices including vegetative stabilized terraces, compost, and legume-cereals crop rotation (LCCR)on a specific plot.

➢Acronym of predictor variables defined in the text.

Results of binary logistic regression in Table 4 verify a prior expectation that factors influence farmers' decision to the combined use of soil conservation practices (CUSCPs). From 12 socioeconomic variables, only productive labor size (PROLABSIZ) and farmland holding size (FARMSIZE) were found to significantly influence the adoption of conservation practices. More productive labor size per household level had a significant effect on farmers' decision to adopt terracing (at p < 0.05) and composting (at p < 0.01). This perhaps implies that a larger potential of productive labor size influences farmers' decision to maintain terraces and to prepare compost than lesser productive labor size on the household level. More productive labor size on the household level determined to prepare compost and to continuously maintain already established terraces as those practices characterized as too labor-intensive (Bewket, 2007; Haregeweyn et al., 2015; Teshome et al., 2016; Mengistu and Assefa, 2019). The unwillingness of farmers to regularly maintain and stabilize the practice due to inadequate productive labor size result in ineffectiveness in reducing run-off, and losses of soil, water, and added nutrients. This further results in more limited short-term economic benefits of ecosystem services in the humid sub-tropic areas (Mekuriaw et al., 2018). The situation may worsen for farmers holding small farmland size with low productive labor size. This may require pertinent technologies to simplify the labor-intensive nature of composting, and terraces construction and maintenance for lesser landholders with low productive labor to intensify their farmlands. The effect of total farmland holding size per head was found to have negative significance at P < 0.05 on the adoption of composting, and positive significance at P < 0.01 to practice LCCR. This implies that lesser farmland holders are more likely to invest a substantial amount of labor in improving soil fertility by using compost. Belay and Bewket (2013) reported that farmers with less landholding were more likely to use compost compared to larger landholder farmers. On the contrary, small farmland holders were less likely to rotate legume-cereals crops and its overturn on the same cultivated fields frequently. The main concern of small farmland holders is to produce major staple food crops like cereals on small own fields for their survival rather than afford to alternated with legume crops repeatedly (Tegegne, 1998). Moreover, despite the coefficients of farmland size is positive on the adoption of terraces and negative on using CUSCP, its effect was not statistically significant.

As expected, institutional factors including extension contact (EXTN) and ownership status of croplands (FLOWNS) were influenced farmers' adoption decision of introduced soil conservation practices. Extension contact was influenced farmers' decision to adopt compost and terraces in cultivated fields respectively at P < 0.01 and P < 0.05 significant levels. This notifies that farmers who had substantial extension contacts more likely to use compost and maintain terraces than farmers that had fewer extension contacts. The implication is that the importance of extension as a source of information and capacity building for smallholder farmers. This is perhaps due to composting, and maintaining of terraces stabilized with the growth of vegetation are characterized relatively as management skill-intensive. Better access to extension services could influence the level of improved soil conservation efforts (Anley et al., 2007; Kassie et al., 2009; Haregeweyn et al., 2015; Mekonnen et al., 2016; Meseret and Amsalu, 2017; Asmame and Abegaz, 2017; Mekuriaw et al., 2018; Melese et al., 2019; Mengistu and Assefa, 2019). Ownership status of cultivated fields (FLOWNS) was influenced positively farmers' decision to adopt terracing, and to CUSCPs on a specific plot at P < 0.01 significant levels. This implies that owners are more likely adopted terracing and CUSCPs realizing that long-horizon planning than cash and share renters. Kassie et al. (2009) reported that ownership of the plot had a positive effect on farmers’ decision to use compost and to combine compost and conservation tillage. Ownership status could increase the assurance of future access to the returns of investments (Aljerf, 2018). Renters may not be interested in maintaining practices in curbing soil loss and replenishing nutrient stock as the pay-off is not always directly visible (Haregeweyn et al., 2015). They are not often considered long term ecological benefits to farmland productivity rather than short-term gains (Perrings, 2014).

Sustainable agricultural production systems are instinctively site-specific within particular inclusive of plot-level and technical perspective attributes (Lee, 2005). From the six hypothesized field level and technical predictor variables, four had significant effects on farmers' decisions (Table 4).

For instance, farmers' perceived plot distance from residence (PERDIST) had positive and significant effects on farmers’ decision to adopt terracing (at P < 0.01) and compost (at P < 0.05), to use LCCR (at P < 0.01), and CUSCP on a particular plot (at P < 0.05). This notifies that more husbandry intensification makes with a decrease of plot distance from farmers' residences. The implication that the probabilities of integrated use of different components of soil conservation practices are increasing with decreasing farmland distance from home. Farmers could manage their farmland according to their perceived closeness. Perhaps, farmers who hold farmlands nearby residences more likely to invest intense management efforts with fewer costs and inputs. This may be due to farmers' perception of the uncertainty of farmland security, labor difficulty transporting compost, and inaccessibility to control *Sesbania Susban* stabilized terraces. The intensity of soil management was increased with decreasing distance from the residential area (Tittonell et al., 2005; Anley et al., 2007; Belay and Bewket, 2013; Mekonnen et al., 2016; Melese et al., 2019). The perceived fertility status of croplands (PERFERT) had a positive significant effect on CUSCPs (p < 0.05) at a particular plot. This implies that CUSCP is more likely to increase when farm plots perceive to be infertile. Plots with infertile soils had positive significant effects on farmers' adoption and continued use of stone terraces (Amsalu and De Graaff, 2007). This suggests that not realizing the short-run negative effect of erosion. However, despite the coefficients of the perceived infertile status of plots were negative on the adoption of compost and positive on the practice of LCCR, effects were not statistically significant.

Farmers' perception of technical fitness of structural practices (TECHFIT) had a positive significant effect (at P < 0.01) in practicing terracing and CUSCPs on a specific plot. Appropriate technical design and effectiveness of terraces in reducing runoff and sediment yield enables the adoption likelihood of CUSCPs on a specific plot. The combined use of vegetation stabilized and technically fitted soil bunds with compost under the legume-cereal crop rotation is the best alternative approach to sustainable cropland management (Debie, 2020). A positive attitude towards the technical fitness of physical conservation practices further encourages the combined use of more conservation practices between terraces (Bijania et al., 2017). The technical fitness of introduced conservation technologies to farmers' requirements and farming system circumstance is one main encouraging factor to the sustainable adoption and widespread replication of the practices (Bewket, 2007). Although farmers are well-aware of the problem of soil erosion, their adoption to introduce soil conservation practices is limited due to inappropriate technical fattiness to the particular plot character (Haregeweyn et al., 2015).

### 4.2 The combined use of conservation practices for sustainable agricultural production

Depending on site-specific conditions, there needs to decide on the combined use of soil erosion controlling and nutrient management practices supplemented with planting vegetation for fodder and fuel-wood (Almaw et al., 2019). The combined use of vegetation stabilized terraces, compost and legume-cereal crop rotation on a specific field was significantly (at P < 0.01) determined by the technical fitness of constructed terraces and ownership status of the cultivated land. Besides, farmers' awareness of the fertility status of the cultivated field, and field distance from the residence were influenced at P < 0.05 significant levels. In the study watershed, farmers gave priority to crop yield-enhancing, followed by reducing soil loss/damaging of crop seedlings and reducing costs of inorganic fertilizers, and improving fodder production (Debie, 2016).

In addressing those benefits, farmers highly preferred the combined use of reafforestation stabilized and technically fitted terraces and compost under the legume-cereal crop rotation system in a particular cultivated field (Debie, 2020). Nutrients use efficiency and water use efficiency are effectively protected in the fields where terraces are well-constructed, stabilized with vegetative practices, properly maintained, and complemented with appropriate practices of drainage ditches (Adimassu et al., 2017; Subhatu et al., 2017; Gathagu et al., 2018; Debie et al., 2019). In the minimum tilled field, the inclusion of crop residues under legume-cereal crop rotation significantly improved crop yield (Monica et al., 2019). This could be more efficient for sustaining high crop yield with low chemical fertilizer requirement when supplemented with the application of compost (Qin et al., 2015; Monica et al., 2019). Planting multipurpose grasses and trees on properly constructed soil bunds for fodder or fuel-wood harvesting could offset crop yield loss owing to the area occupied by the bunds (Adimassu et al., 2017; Debie et al., 2019).

In addition to economic contributions, the combined use of conservation practices could address climate change mitigation and other ecosystem services. The inclusion of crop residue in the legume-cereal rotation system combined with compost application sequester soil organic carbon (Poeplau and Don, 2015). Soil carbon represents 25% of the potential of natural climate solutions, of which 40% is the protection of existing soil carbon and 60% is rebuilding depleting stocks (Bossio et al., 2020). Building soil carbon is an appealing way to reduce carbon emissions owing to soil degradation and crop production (Hellin and Fisher, 2019; Bossio et al., 2020). Thus, sustainable management of the farming system through integrating protective, and nutrient additive conservation practices feed growing populations while reducing greenhouse gas emissions and conserving natural resources (Hellin and Fisher, 2019).

## 5 Conclusions

Smallholder farmers' decision to adopt/use vegetation stabilized terraces, compost, legume-cereals crop rotation (LCCR), and combined use of these conservation practices (CUSCPs) on a specific plot was influenced by intermingled factors. For instance, farmers' decisions to adopt and maintain terraces were influenced significantly by productive labor size, extension contact, plot distance from home, and technical fitness of terraces. Decision at farm household level to use compost in any farmland was influenced positively and significantly by productive labor size and degree of extension contact, while influenced negatively and significantly by total farmland holding size (in ha). Farmers' decision to practice LCCR was explained positively and significantly by farmland holding size (in ha), farmland distance from home, and perceived soil fertility status of a particular plot. Besides, the ownership status of farmlands, plot distance from home, fertility status, and technical fitness of terracing were the major determinants for farmers’ decision to CUSCPs on a specific plot. Therefore, agricultural extension systems should recognize these factors to influence farmers' decision to adopt overtime and scale-up multifunctional CUSCPs in the broad context for the sustainable agricultural production of smallholder farmers.

## Declarations

### Author contribution statement

E. Debie: Conceived and designed the experiments; Performed the experiments; Analyzed and interpreted the data; Contributed reagents, materials, analysis tools or data; Wrote the paper.

### Funding statement

This research did not receive any specific grant from funding agencies in the public, commercial, or not-for-profit sectors.

### Data availability statement

Data will be made available on request.

### Declaration of interests statement

The authors declare no conflict of interest.

### Additional information

No additional information is available for this paper.
